# Porous Glass with Layered Morphology Prepared by Phase Separation

**DOI:** 10.3390/ma18051133

**Published:** 2025-03-03

**Authors:** Qiang Chen, Yihong Chen, Zhe Wang, Yao Zhou, Changjiu Li

**Affiliations:** 1 State Key Laboratory of Marine Resource Utilization in South China Sea, Hainan University, Haikou 570228, China; 21210805000002@hainanu.edu.cn (Q.C.); 18751961263@163.com (Y.C.); wangzhe@hainanu.edu.cn (Z.W.); yao_zhou@hainanu.edu.cn (Y.Z.); 2 College of Materials Science and Engineering, Hainan University, Haikou 570228, China

**Keywords:** lamellar, microphase, phase separation, sodium borosilicate glass

## Abstract

In this study, porous glass with controllable layered structure was successfully prepared by the phase-separation method, with the aim to develop a high-performance high-temperature catalytic (denitrification) material. Glass compositions with different R values (n (Na_2_O)/n (B_2_O_3_)) were designed based on the phase diagram of sodium borosilicate glass. The layered porous structure was obtained by heat treatment in the phase-separation temperature range and acid-leaching treatment to remove the boron-rich phase. For the adsorption and separation process, the layered pore is very ideal, due to its high contact area, high storage capacity and easy mass transfer characteristics, which means it has high adsorption capacity and separation efficiency. The experimental results show that the thickness of the silicon layer can be precisely controlled in the range of 2–23 μm by adjusting the heat treatment time (1.25–10 h), and the material has excellent high-temperature stability (the pore structure parameters do not change significantly after calcination at 600 °C for 10 h). V_2_O_5_ (multiphase redox catalyst) can be uniformly loaded by the impregnation method, and the layered structure can be completely retained. The formation process of the layered structure was studied by infrared, Raman spectroscopy and SEM analysis. This study provides a new strategy for the development of customizable porous materials.

## 1. Introduction

With the continuous advancement of global industrialization, the problem of air pollution is becoming more and more serious, and nitrogen oxides (NOx) are one of the main air pollutants. In order to reduce the emission of nitrogen oxides, denitration treatment before plant gas emission has become a necessary environmental protection measure. Many researchers have conducted in-depth research on denitration catalysts and developed various types of catalysts. These include noble metal catalysts such as Pt, Ag, Ru, Pd [1,2,3], and Au and metal oxide catalysts such as V_2_O_5_, CeO_2_, Fe_2_O_3_ [4,5,6,7]. The activity of the catalyst mainly depends on its particle size and dispersion [8]. A good catalyst support material can provide good dispersion, effective contact surface area and use a small amount of catalytically active components [9].

Common high-temperature denitrification carrier materials include porous TiO_2_, γ-Al_2_O_3_, SiC, and porous glass [10,11,12,13,14,15]. Porous TiO_2_ has excellent catalytic performance below 450 °C, but its thermal stability at high temperature is poor, which can easily lead to structural changes, thus affecting the catalytic effect [16]. γ-Al_2_O_3_ has a weak ability to resist sulfur poisoning. In the flue gas environment containing SO_2_, the surface of γ-Al_2_O_3_ easily adsorbs SO_2_ and forms sulfate, which covers the active sites and inhibits the adsorption and reaction of NH_3_ [17]. With high specific surface area and excellent durability, porous SiC can stably perform catalytic reactions in harsh environments, especially in high-temperature air, showing excellent structural stability [10]. However, SiC materials are relatively scarce, and it is difficult to obtain and process. In contrast, the thermal stability of porous glass is better than that of TiO_2_, the resistance to sulfur poisoning is higher than that of Al_2_O_3_, and the material source is wider than that of SIC. Porous glass has the advantages of high specific surface area, good thermal stability and a wide range of material sources, and is a kind of denitration carrier material with broad application prospects.

The main component of porous glass is silica (SiO_2_), and the content is usually more than 96%. Porous glass has uniformly distributed nano-scale or micro-scale pores. These pores give it the advantages of high specific surface area and high pore volume. In addition, porous glass has good morphological controllability, chemical stability and high thermal stability [18,19]. These characteristics give porous glass broad application potential in the fields of high-temperature catalysis, adsorption, and biosensors. Porous materials with ordered and controllable mesoscopic pore structure have shown greater application potential in the field of catalysis and adsorption, and their preparation methods have also become a research hotspot [20]. For the adsorption and separation process, the layered porosity is very ideal, due to the high contact area, strong storage capacity, and easy mass transfer, which lead to high adsorption capacity and separation efficiency. The main methods for preparing layered porous glass are the hard template method and soft template method. The soft template [20,21,22,23,24] method regulates the structure of layered porous glass by adjusting the synthesis parameters such as temperature, pH value, precursor solubility, etc., which is a relatively convenient method. However, the soft template method requires the use of macromolecular block copolymers as soft templates, which are often non-commercial, non-renewable and expensive. The hard template method [25] has the problem that the structure cannot be regulated, and the structure is limited by the template used. The phase-separation method is favored for its simple process and easy availability of raw materials.

This study attempts to prepare layered porous glass with controllable layer thickness and pore size by the phase-separation method by designing sodium borosilicate glass with different compositions, which has high-temperature stability and can be used for high-temperature catalysis. The formation mechanism of layered porous glass structure was studied. These studies not only reveal the microstructure of borosilicate glass, but also provide new insights into the preparation of porous materials with specific structures.

## 2. Experiment

### 2.1. Glass Preparation

Combined with the ternary phase diagram of sodium borosilicate glass, sodium borosilicate glasses with different R values were prepared by using SiO_2_ (99.5%), H_3_BO_3_ (99 %) and Na_2_CO_3_ (99.5%) as raw materials in the phase-separation region. There are basic structural units of [BO_3_], [BO_4_] and [SiO_4_] in sodium borosilicate glass, and the molar ratios of R = n (Na_2_O)/n (B_2_O_3_) and K = n (SiO_2_)/n (B_2_O_3_) are usually used as composition parameters. The R value is crucial for understanding the properties of the glass structure. The ratio of [BO_3_] to [BO_4_] in the glass network can be changed by adjusting the R value. The ratio of [BO_3_] to [BO_4_] determines the group structure formed by [BO_3_] and [BO_4_] in the glass. The compositions of glasses prepared in this study are detailed in Table 1.

The raw materials were mixed by planetary ball mill (DECO, Changsha, China) for 2 h. To minimize volatilization of boron, an additional 10% of boron was added and held at 1550 °C for 1 h. The glass was cast in a steel mold and quickly transferred to an annealing furnace at 350 °C for 5 h, and after that cooled down with ~30 K/h to room temperature.

### 2.2. Phase Separation, Acid Leaching

The glass was cut into 8 × 8 × 3 mm samples. The A2 samples were kept at 600 °C for 1.25 h, 2.5 h, 5 h and 10 h, respectively, and another group of samples were heat-treated at 600 °C for 5 h. The samples after heat treatment were corroded in 2 mol/L hydrochloric acid at 90 °C for 1 h to remove the borate-rich phase. After corrosion, the samples were soaked in deionized water, then dried at 200 °C for 2 h to obtain the final samples (PG).

### 2.3. Loading V_2_O_5_

The oxalic acid solution was mixed with ammonium metavanadate solution, and enough acid was added to adjust to pH = 1, and the solution was allowed to stand for 1 h. The color of the solution was changed to blue, and the porous sample was added. The solution and the sample were moved to a vacuum-drying chamber and immersed in a negative vacuum for 1 h. Then, the sample was dried for 12 h in a 120 °C drying oven, and the sample was taken out and placed in a muffle furnace (Kejing MTI, Hefei, China). The sample was calcined at 550 °C for 6 h to obtain the loaded sample.

### 2.4. Characterization Techniques

The DSC test was performed using Netzch STA 449 F5 Jupiter (Netzsch, Selb, Germany). The prepared glass samples were poured into the mortar and ground into powder. An amount of 25 mg was heated from 24 °C to 800 °C at a rate of 10 °C/min under nitrogen flushing gas (50 mL/min). The characteristic temperature was determined according to the DSC curve. The samples after heat treatment were analyzed by X-ray diffraction (Rigaku, Tokyo, Japan). The XRD spectra were recorded from 10° to 80° with a step of 0.020°. The infrared spectrum test was carried out by KBr dilution method, with KBr as the background, and each sample was scanned 10 times. Infrared test sample preparation, potassium bromide and the sample were mixed evenly in an agate mortar, and pressed for one minute at 8 Tm to obtain a transparent tablet for testing. The structural characteristics of porous glass, such as pore size, pore volume and specific surface area, were analyzed by the BET instrument (Micromeritics, Norcross, GA, USA). and nitrogen adsorption technology. Before the analysis, the samples were vacuum-activated at 250 °C for 10 h and measured at 77 K relative pressure range of 0−0.995 P/P_0_. The morphology of porous glass was measured by scanning electron microscopy (SEM) (Thermo Fisher Scientific, Waltham, MA, USA). In order to ensure sufficient conductivity, the sample was covered with a layer of gold. Under the electron beam irradiation of an ordinary electron microscope, phase separation may occur in glass samples. In order to avoid the influence of electron beam on the structure of the sample, it is recommended to minimize the exposure time of the glass sample in the electron beam of the ordinary electron microscope [26,27]. The energy dispersive spectrometer (EDS) detector (Thermo Fisher Scientific, Waltham, MA, USA) was used for element and mapping imaging, and the excitation energy was 15 keV. The morphology of porous glass was analyzed by electron microscope image. All samples were tested at room temperature.

## 3. Results and Discussion

### 3.1. Phase Separation of Glass

**Figure 1:**
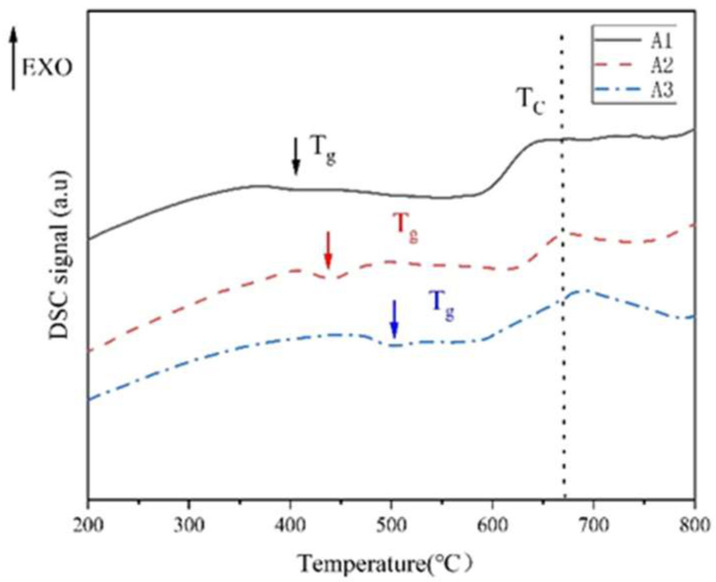
DSC diagram of A1, A2 and A3 element samples.

The phase-separation range in the original glass was determined by the DSC method. As shown in Figure 1, the temperature corresponding to the first endothermic peak is the glass transition temperature T_g_. T_L_ is a double-node (critical) temperature, and the phase-separated glass is mixed again at this temperature point. The phase-separation temperature range is between T_g_–T_L_, and 600 °C is selected as the heat treatment temperature. Through the DSC diagram of A1, A2 and A3 samples, it can be seen that the glass transition temperature and double-node temperature of the samples change monotonously with the R value. When R ≤ 0.3, T_g_ increases from 400 °C to 500 °C. The formation of [BO_4_] and Si-O-B structure in sodium borosilicate glass is the main reason for the increase in glass transition temperature [28]. The increase in R value leads to the increase in free oxygen, the increase in [BO_4_], the more stable glass network structure and the increase in glass transition temperature.

**Figure 2:**
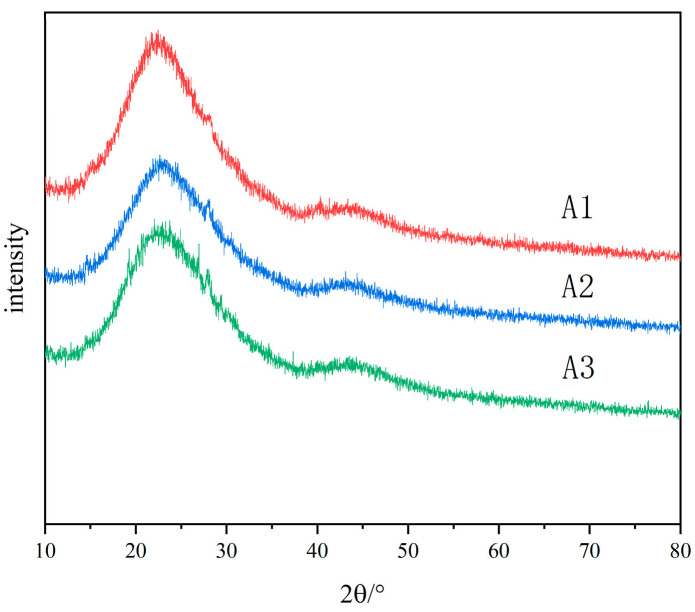
XRD patterns of A1, A2 and A3 heat-treated samples.

The original glass is colorless and transparent. After heat treatment, A1 and A2 samples show white opacities, and A3 samples show slight blue opacities and remain transparent. The samples after heat treatment were analyzed by X-ray diffraction. Figure 2 shows the X-ray diffraction patterns of A1, A2 and A3 samples after heat treatment at 600 °C for 5 h. It shows that the increase in R value strengthens the connectivity of the glass network, slows down the phase-separation trend of the glass, and increases the stability of the glass. The two main peaks observed in the XRD pattern are located at 2θ = 22° and 2θ = 45°, corresponding to B_2_O_3_ and SiO_2_, respectively, which are the common characteristics of glass containing mixed-network-forming bodies. There is no crystal precipitation in the samples of each component after heat treatment, and the opaque glass is caused by phase separation.

### 3.2. Morphology of Phase Separation

**Figure 3:**
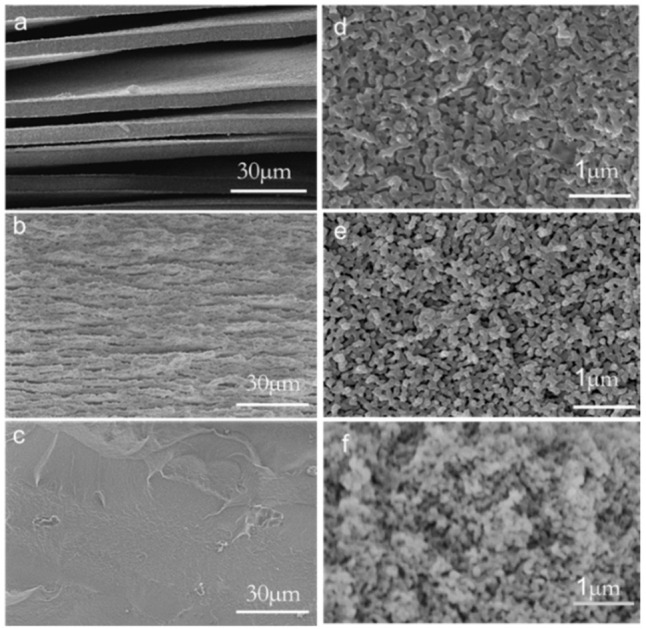
SEM images of PGA1, PGA2 and PGA3 samples after heat treatment at 600 °C for 5 h. The SEM images of (**a**–**c**) are magnified 1000 times, and the SEM images of (**d**–**f**) are magnified 20,000 times.

**Figure 4:**
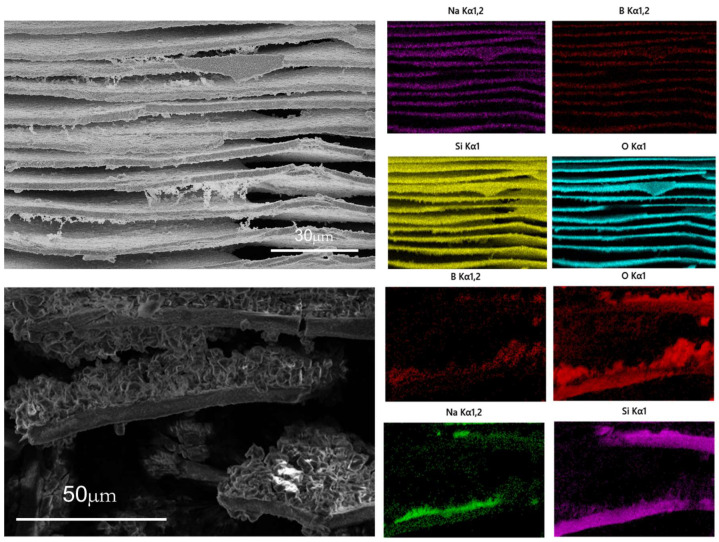
EDS diagram of A2 sample after phase separation and leaching for 60 min (**above**). EDS diagram of A2 sample after leaching for 5 min (**below**).

Samples with varying R values, after being heat-treated at 600 °C for 5 h, were acid-leached, and the SEM images of the resulting glass samples are depicted in Figure 3. From Figure 3a,b, it can be seen that the PGA1 and PGA2 formed a more obvious layered structure from the macroscopic structure after leaching. From Figure 3d,f, it can be seen that the layer is composed of a worm-like structure with a large number of pores. Figure 3c shows that after leaching treatment, PGA3 did not exhibit layering phenomena on a macroscopic level, and the overall structure remained intact. Figure 3f indicates that the surface of PGA3 also displayed a worm-like structure. The sample phase separates the silicon-rich phase and the sodium-rich boron phase. The sodium-rich boron phase and the silicon-rich phase form a bicontinuous phase. After leaching, the sodium-boron phase dissolves, leaving the silicon-rich phase. The worm-like structure is mainly composed of amorphous silica, which can also be proved by EDS (Figure 4). From samples (d), (e), and (f), it can be observed that as the R value increases, the leached silica skeleton becomes denser, and the average width of the silica decreases. The bicontinuous phase formed by the sodium boron phase and the silicon-rich phase is finer and denser, indicating that the R value increases and the phase-separation degree of the sample decreases.

Figure 4 is the EDS diagram of A2 sample after heat treatment at 600 °C for 5 h and leaching at 90 °C for 60 min and 5 min with 2 mol/L hydrochloric acid. After 60 min of leaching, the sample is a layered structure, and the content of silica in the layered structure accounts for more than 96%, with only a small amount of sodium and boron remaining. After leaching for 2 min, it was found that there were incompletely leached samples in the middle of the silica layer. EDS elemental analysis of the samples showed that the silicon element accounted for the main component in the layered structure, and the elements of the interlayer samples were mainly sodium and oxygen element boron.

### 3.3. Thermal Stability of Glass

The layered porous glass obtained by heat treatment of A2 sample at 600 °C for 5 h was calcined at 600 °C for 10 h to test its thermal stability (PGA2-600). The pore structure parameters of porous glass before and after calcination were measured by BET. The pore structure parameters of the samples before and after calcination were basically unchanged, which indicates that the porous glass has good thermal stability (Table 2).

### 3.4. Catalyst Loading Morphology

The thickness and pore size of the silica layer significantly affect the adsorption capacity and separation efficiency of sodium borosilicate glass in catalytic applications by affecting the specific surface area, pore volume and mass transfer path. In this study, the change in aperture is small. For the PGA2 sample, the thinner layer can provide higher specific surface area and pore volume, and the adsorption capacity and separation efficiency are stronger. PGA2 was loaded with vanadium pentoxide by the impregnation method.

**Figure 5:**
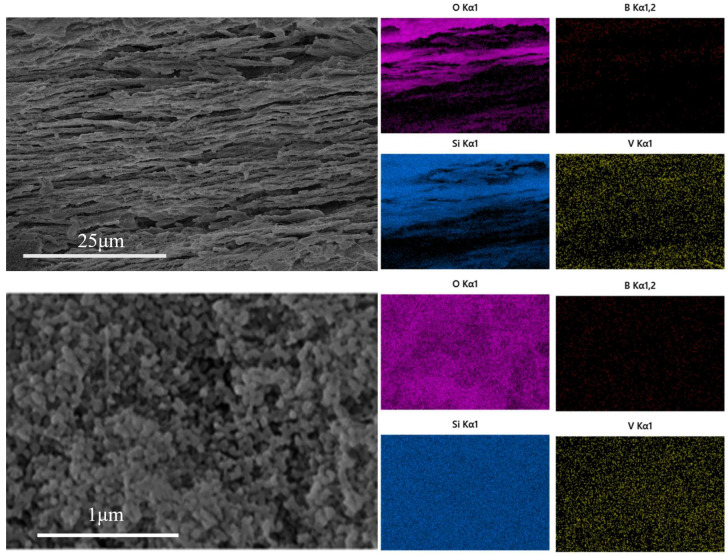
EDS diagram of vanadium pentoxide-loaded porous glass.

As shown in Figure 5, vanadium pentoxide is uniformly loaded in the porous glass. The layered structure of the porous glass remains intact, and the pore structure is not destroyed. The uniformly distributed V_2_O_5_ can effectively increase the exposed area of the catalytic active sites and promote the adsorption of reactants through surface oxygen vacancies and acidic sites. In addition, the uniform load also avoids the local aggregation of V_2_O_5_ at high temperature [29,30,31].

### 3.5. Mechanism Analysis

#### 3.5.1. Structural Analysis

**Figure 6:**
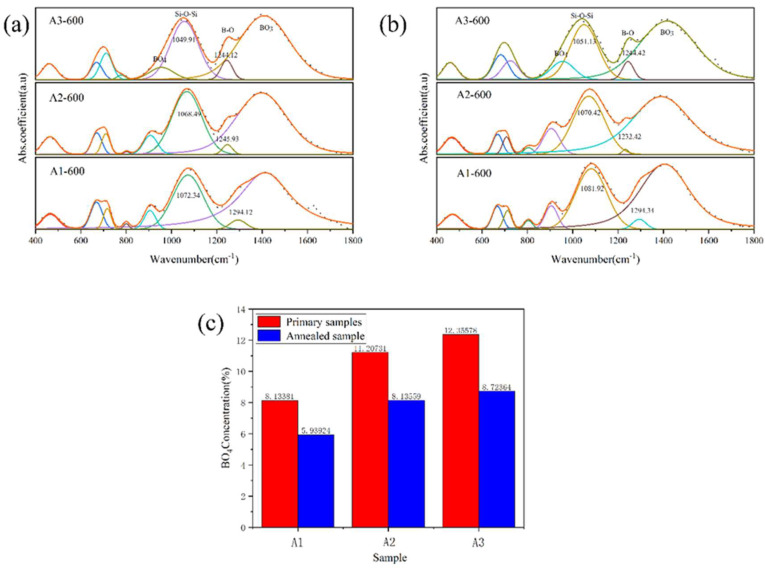
The fitting infrared spectra of A1, A2 and A3 samples before phase separation (**a**) and after phase separation (**b**), and the integral area ratio of [BO_4_] and [BO_3_] before and after phase separation (**c**).

Through infrared spectroscopy analysis, we studied the structural differences between different samples and the structural changes before and after phase separation. The complete data are shown in Figure S1. The fitted data are shown in Figure 6. Table 3 provides the distribution of infrared spectral vibration modes of sodium borosilicate glass by previous researchers.

With the increase in R value, the peak position of Si-O-Si asymmetric stretching vibration shifted from 1081 cm^−1^ to 1051 cm^−1^. The infrared vibration of Si-O-Si depends on the average bond angle of Si-O-Si, and the decrease in wavenumber means that the average bond angle of Si-O-Si decreases. After the phase separation of the sample, the Si-O-Si asymmetric stretching vibration band also shifts to a low wavenumber. For the wave bands (460,800 cm^−1^) caused by Si-O-Si vibration, the strength decreases with the increase in R value. The fluctuation bands of 920 cm^−1^ and 1400 cm^−1^ correspond to the boron–oxygen tetrahedron [BO_4_] and the boron–oxygen triangle [BO_3_] in the glass, respectively. With the increase in R value, the area integral ratio of the wave bands at 920 cm^−1^ and 1400 cm^−1^ increases, and the vibration band of the asymmetric stretching vibration of the B-O bond shifts to the low wavenumber. With the increase in R value, the free oxygen content in the glass increases, [BO_3_] is converted to [BO_4_], the connectivity of the glass increases, and the trend of glass phase separation slows down. After the phase separation of the sample, the area integral ratio of the wave bands at 920 cm^−1^ and 1400 cm^−1^ decreases. The phase separation promotes the transformation of [BO_4_] to [BO_3_] in the glass, and the structure of the boron in the glass changes from the shelf structure to the layered structure. The area integral ratio of [BO_4_] and [BO_3_] is shown in Figure 6c.

**Figure 7:**
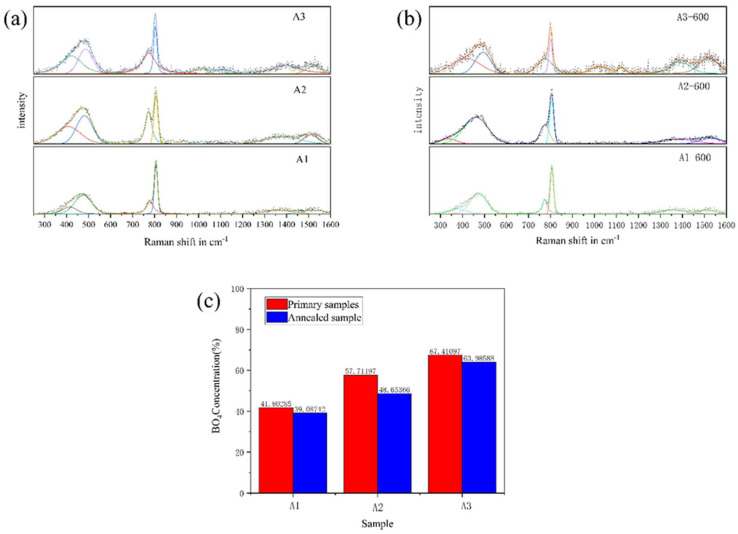
The fitted Raman spectra of A1, A2 and A3 samples before phase separation (**a**) and after phase separation (**b**), and the ratio of the integral area of [BO_4_] and [BO_3_] in the boron oxide ring before and after phase separation (**c**).

Raman tests were performed on A1, A2, and A3 samples before and after phase separation. The block sample was used for testing, and the surface of the sample was polished before the test. The fitting results are shown in Figure 7, and the complete data are shown in Figure S2. Furthermore, summarized deconvoluted Raman bands assignment is provided in Table 4.

It can be seen from Figure 7 that there are five main vibration peaks in the Raman spectra of A1, A2 and A3 samples before phase separation. It can be seen from Table 3. The peaks in the 300–600 cm^−1^ band are related to the mixed stretching and bending vibration modes of Si-O-Si, B-O-B and B-O-Si bonds. The peak at 805 cm^−1^ corresponds to the three-coordinated boron in the boroxol ring, while the peak at 780 cm^−1^ corresponds to the four-coordinated boron in the boroxol ring (asymmetric boroxol ring). The peak at 1360 cm⁻¹ is related to the stretching vibration of the non-cyclic [BO₃] units connecting to the [BO₄] unit. The peak at 1515 cm⁻¹ is attributed to the tensile vibration of the [BO₃] unit in the boroxol ring. The tensile vibration of Si-O-Si appeared in the A3 sample at 900–1200 cm^−1^, indicating that the free oxygen in the A3 sample was combined with silicon. With the decrease in R value, the peak areas of 780 cm^−1^ and 808 cm^−1^ are gradually enhanced, indicating that the content of boron oxygen ring in sodium borosilicate glass is increasing, so the newly added boron oxide is added to the glass network in the form of boroxol ring. The ratio of 780 cm^−1^ and 808 cm^−1^ peak area in the original samples of A1, A2 and A3 increased with the increase in R value. The increase in R value promotes the content of asymmetric boroxol ring. The main characteristic peaks of Raman spectra of the samples before and after phase separation remain unchanged. In the samples after phase separation, the peak area at 780 cm^−1^ decreases and the peak area at 805 cm^−1^ increases. The glass forms a sodium-rich boron phase and a silicon-rich phase after phase separation, and the borate structure mainly exists in the sodium-rich boron phase. After phase separation, there are more symmetrical boron oxide rings in the sodium-rich boron phase, the asymmetric boron oxide ring is reduced, and the connectivity of the sodium-rich boron phase is reduced.

#### 3.5.2. Evolution of Phase-Separation Structure with Time

**Figure 8:**
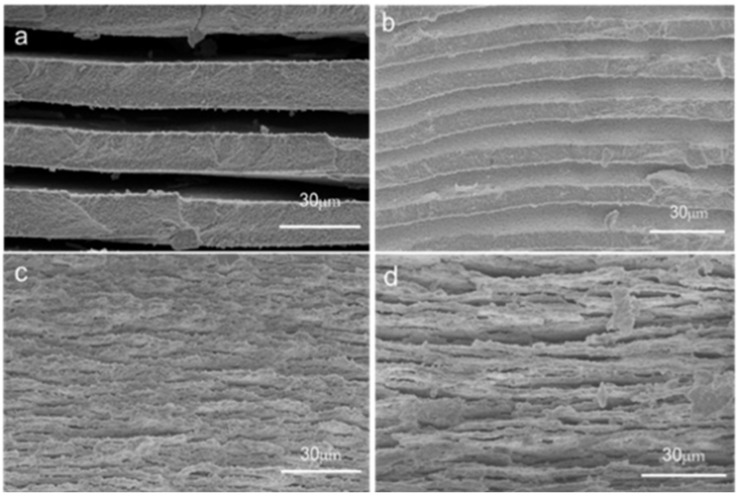
Low-power scanning electron microscopy of A2 samples with different heat treatment time: (**a**) 1.25 h, (**b**) 2.5 h, (**c**) 5 h, (**d**) 10 h.

**Figure 9:**
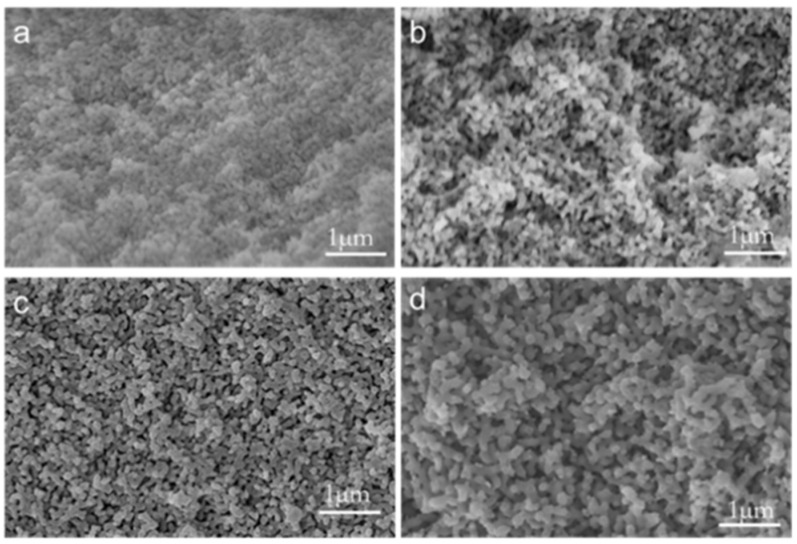
A2 sample 600 °C heat treatment at different times of high-power scanning electron microscopy (**a**) 1.25 h, (**b**) 2.5 h, (**c**) 5 h, (**d**) 10 h.

Figure 8 and Figure 9 are the low- and high-magnification scanning electron microscope images of PGA2 samples with different heat treatment time. In Figure 8a–d are the low-power scanning electron microscope images of the porous glass obtained by A2 sample after heat treatment at 600 °C for 1.25 h, 2.5 h, 5 h and 10 h, respectively. It can be seen from Figure 6 that all the samples show a macroscopic layered structure. The thickness of the layered structure can be obtained by measurement software (Nano Measurer 1.2.5) analysis, and the measurement results are shown in Figure S3. When the heat treatment time is 1.25 h at 600 °C, the thickness of the layered structure is the highest, which can reach 23 microns. The thickness of the layered structure is reduced to the minimum of 2 microns after heat treatment at 600 °C for 5 h. The thickness of the layered structure of the samples heat-treated for 5 h and 10 h has almost no change. The thickness of the layered structure changes significantly under different heat treatment time.

Figure 9a–d are the high-power scanning electron microscope images of porous glass obtained by leaching A2 samples after heat treatment at 600 °C for different time. It can be seen from the figure that the surface of the samples with different heat treatment time showed similar morphological phases after leaching. All samples are stacked by worm-like silica. Through the analysis of the measurement software (Nano Measurer 1.2.5), the average width of the silica phase can be obtained. As the temperature increases, the average width of the silica phase increases from 0.08 μm to 0.17 μm.

#### 3.5.3. Mechanism of Layered Phase Separation

After the heat treatment of sodium borosilicate glass, the sodium borosilicate glass undergoes spinodal phase separation. The silicon-rich phase and the sodium-rich boron phase form a bicontinuous structure. During the phase-separation process, the chemical composition of the sodium-rich boron phase and the silica-rich phase changes with time until the equilibrium composition is reached. After the glass phase separation, the corrosion can form a uniform silicon dioxide layer (see Figure 3), and there is a sodium-rich boron phase layer between the silicon dioxide layers (see Figure 4). The structure of sodium borosilicate glass was analyzed. After phase separation, the content of [BO_4_] in the sodium-rich boron phase decreased, the symmetrical boroxol ring increased, and the asymmetric boroxol ring decreased. The boron structure in the sodium-rich boron phase is mainly a layered structure. These structural changes are conducive to the formation of sodium boron phase layer (see Figure 6 and Figure 7).

With the passage of time, the results of phase separation show that the silica layer gradually becomes thinner, while the silica phase particles gradually increase (see Figure 8 and Figure 9). The growth of these particles is a result of the subsequent stage of phase separation, which effectively reduces the interfacial energy of the system and thereby enhances the overall thermodynamic stability. In the process of phase separation, the sodium-rich boron phase is gradually separated from the glass matrix, and its volume increases gradually, while the volume of the silicon-rich phase decreases accordingly. The silicon-rich phase must shrink to ensure that the silica particles can continue to grow while the volume of the silicon-rich phase decreases. The rigidity of the silicon–oxygen bond limits the fluidity of the silicon-rich phase. The silicon-rich phase cannot move on the macroscopic scale, so that it is still a bicontinuous structure after long-term phase separation.

**Figure 10:**
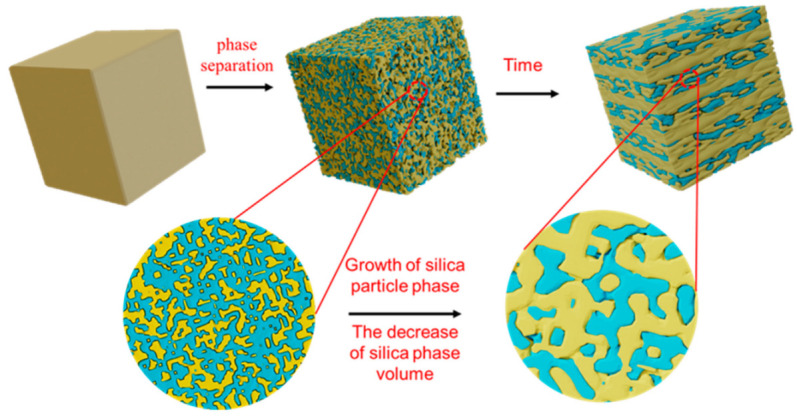
Evolution of layered structure with time.

With the continuous shrinkage of the silicon-rich phase during the phase-separation process, these silicon–oxygen bonds eventually break each other to form a uniform silicon-rich layer. The excess sodium-rich boron phase was squeezed out and aggregated, which contained a large amount of [BO_3_] layered structure, and finally formed a sodium-rich boron layer. With the further development of phase separation, more sodium-rich borosilicate layers are formed, and a large number of silica layers are also formed in the glass. This process not only reveals the dynamic change in phase separation, but also shows the evolution of material microstructure. Figure 10 shows this evolution process. The glass phase is separated to form a silicon-rich phase (blue) and a sodium-rich boron phase (yellow).

The reason for the failure of A3 sample to form a layered structure is that the increase in R value (i.e., n (Na_2_O)/n (B_2_O_3_)) leads to the increase in free oxygen content in the glass, which, in turn, promotes the conversion of [BO_3_] to [BO_4_]. This transformation process enhances the connectivity of the glass network. The stabilization of this network inhibits the kinetic process of phase separation, thereby slowing the separation behavior of the silicon-rich phase and the sodium borocalcite phase.

## 4. Conclusions

In this study, we prepared layered high silicon glass with controllable layer thickness and pore size by the phase-separation method. Through experiments, we successfully obtained layered porous glass with a thickness of 2−23 μm, a specific surface area of 60−220 m^2^/g, a pore volume of 0.1−0.3 cm^3^/g, and a pore size of 3−9 nm. The material exhibits excellent high-temperature thermal stability, and its pore structure remains basically unchanged after high-temperature calcination. In addition, the layered porous structure can be completely maintained after uniform loading of V_2_O_5_ by the impregnation method.

During the phase-separation process, the silicon–oxygen bond cannot move on the macroscopic scale due to its inherent rigidity and directivity, resulting in a bicontinuous structure in the results. As the phase separation continues, the silicon-rich phase shrinks and eventually breaks to form a uniform layered structure. At the same time, the excessive sodium-rich boron phase is extruded and aggregated, which contains a large number of planar [BO_3_] structures, and finally forms a sodium-rich boron layer. The layered structure is the result of the evolution of phase-separation morphology. With the decrease in R value, the content of [BO_3_] in the glass increases, which not only promotes the phase separation of the glass, but also contributes to the formation of sodium borosilicate phase layer after phase separation. Therefore, A1 and A2 samples with smaller R values were successfully obtained in the uniform layered porous structure after phase separation and corrosion treatment. In contrast, the A3 sample with a larger R value failed to form a layered structure after the same treatment.

This study not only provides a new strategy for the controllable preparation of layered porous glass, but also clarifies its structure formation mechanism through multi-scale characterization, which lays a theoretical and experimental foundation for its application in high-temperature catalysis, adsorption and other fields.

## Figures and Tables

**Table 1:** Main chemical composition of sodium borosilicate.

Glass	B_2_O_3_	SiO_2_	Na_2_O	R = (Na_2_O/B_2_O_3_)	K = (SiO_2_/B_2_O_3_)
A1	50.25	44.72	5.03	0.10	0.89
A2	48.00	42.80	9.20	0.20	0.89
A3	45.66	40.64	13.70	0.30	0.89

**Table 2:** The pore structure parameters of PGA1, PGA2, PGA3, PGA2-600 samples.

Samples	Average Pore Size of Sample (nm)	Specific Surface Area (m^2^ g^−1^)	Pore Volume (cm^3^ g^−1^)
PGA1	7.11 ± 0.35	68.76 ± 1.38	0.12 ± 0.0012
PGA2	8.88 ± 0.44	149.06 ± 2.98	0.33 ± 0.0033
PGA2-600	8.82 ± 0.44	146.99 ± 2.94	0.32 ± 0.0012
PGA3	3.97 ± 0.20	218.59 ± 4.37	0.21 ± 0.0022

**Table 3:** Distribution of infrared spectral vibration modes of sodium borosilicate glass.

Wavenumber (cm^−1^)	Vibration Types
400–500	Si-O-Si bending vibration [32,33,34,35]
670	Si-O-B bending vibration [34,35,36]
700	[BO_3_] bending vibration [32,33,34,35,36]
800	Si-O-Si symmetric stretching vibration [32,33,34,35,36,37,38,39]
900–1000	Asymmetric stretching vibration of [BO_4_] [32,33,34,35,36,37,38,39,40]
1050–1150	Si-O-Si asymmetric stretching vibration [35,36,37,38,39,40]
1260–1310	The stretching vibration of B-O-B [33,34,35,36,37,38,39]
1400	Asymmetric stretching vibration of [BO_3_] [32,33,34,35,36,37,38,39,40]

**Table 4:** Raman spectral characteristic vibrations common in sodium borosilicate glass.

Raman Shift (cm^−1^)	Raman Assignment
300–600	Mixed bending and stretching of bridged Si-O-Si, Si-O-B and B-O-B in three-dimensional network [41,42,43]
780	Vibration of four-coordinated boron in an asymmetric boroxol ring [44,45,46,47]
808	Vibration of three-coordinated boron in symmetrical boroxol ring [44,45,46,47]
900–1200	Tensile vibration of Si-O-Si (containing 2–4 bridge oxygen) [45,46,47,48]
1360	Stretching of non-annular [BO_3_] linked to [BO_4_] [48,49]
1515	Stretching of three-coordinated boron in boron oxide ring [44,45,46,47,48]

## Data Availability

The original contributions presented in the study are included in the article/Supplementary Materials, further inquiries can be directed to the corresponding authors.

## Supplementary Materials

The following supporting information can be downloaded at: https://www.mdpi.com/article/10.3390/ma18051133/s1, Figure S1: The original infrared images before and after phase separation; Figure S2: The original Raman spectra before and after phase separation: title; Figure S3: Graph of layer thickness at different heat treatment times.
