# Supplementary material for: Porous Glass with Layered Morphology Prepared by Phase Separation
Source: Materials (Basel). 2025 Mar 3;18(5):1133. doi: 10.3390/ma18051133 (PMC11901456; doi:10.3390/ma18051133)
Supplement: Supplementary file 1 [file materials-18-01133-s001.zip › materials-3447913-supplementary.pdf]

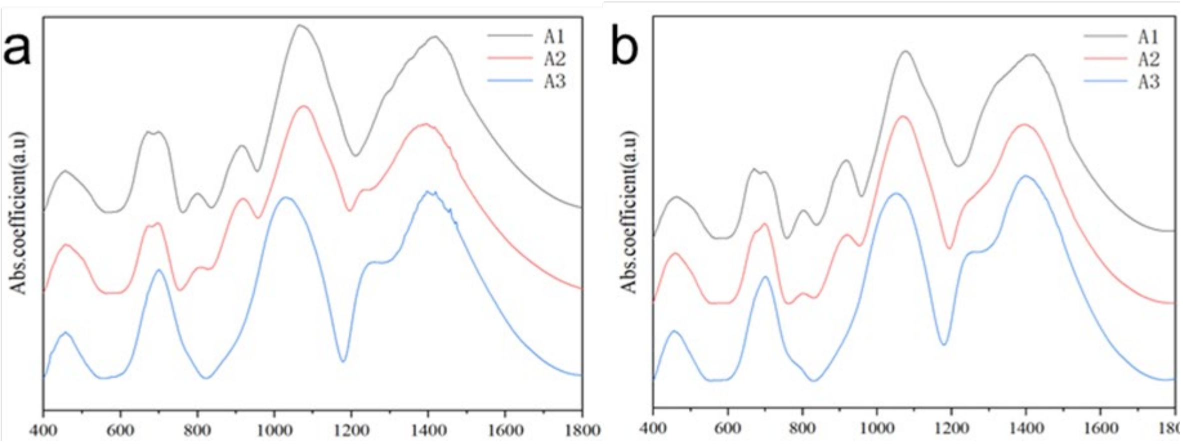

Figure S1. The original infrared images before and after phase separation.

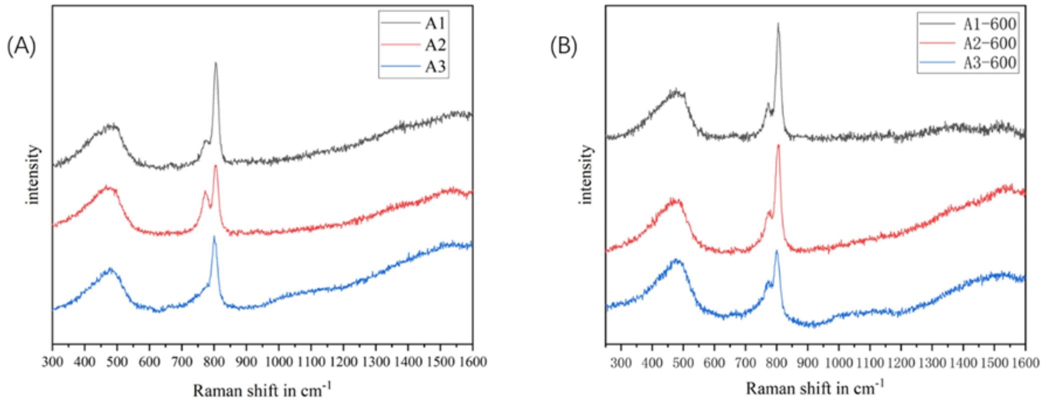

Figure S2. The original Raman spectra before and after phase separation.

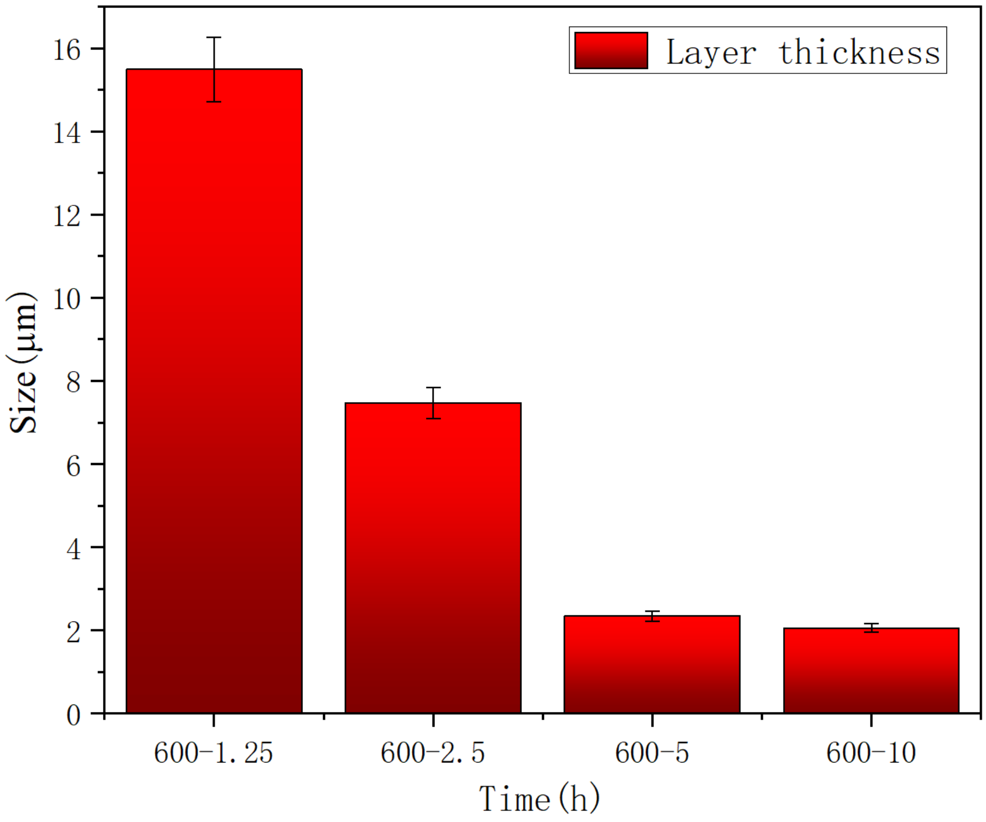

Figure S3. Graph of layer thickness at different heat treatment times.
